# The value of delta neutrophil index in young infants with febrile urinary tract infection

**DOI:** 10.1038/srep41265

**Published:** 2017-02-07

**Authors:** Jung Won Lee, Seong Heon Kim, Se Jin Park, Keum Hwa Lee, Jae Hyon Park, Andreas Kronbichler, Michael Eisenhut, Ji Hong Kim, Jong Wook Lee, Jae Il Shin

**Affiliations:** 1 Department of Pediatrics, Ewha Womans University School of Medicine, Seoul, Korea; 2 Department of Pediatrics, Pusan National University Children’s Hospital, Yangsan, Korea; 3 Department of Pediatrics, Geoje Children’s Hospital, Geoje, Korea; 4 Department of Pediatrics, Yonsei University College of Medicine, Seoul, Korea; 5 Department of Pediatric Nephrology, Severance Children’s Hospital, Seoul, Korea; 6 Yonsei University College of Medicine, Seoul, Korea; 7 Medical University Innsbruck, Department of Internal Medicine IV (Nephrology and Hypertension), Innsbruck, Austria; 8 Luton & Dunstable University Hospital NHS Foundation Trust, Luton, United Kingdom; 9 Department of Laboratory Medicine, Jincheon Sungmo Hosipital, Jincheon, Korea; 10 Research Institute of Bacterial Resistance, Yonsei University college medicine, Seoul, Korea; 11 Institute of Kidney Disease Research, Yonsei University College of Medicine, Seoul, Korea

## Abstract

Delta neutrophil index (DNI) is the fraction of circulating immature granulocytes, which reflects severe bacterial infections and septic condition but has not been studied in urinary tract infection (UTI). Here, we evaluated the value of DNI in predicting acute pyelonephritis (APN) or vesicoureteral reflux (VUR) using the data of 288 patients. Conventional inflammatory markers (white blood cell [WBC] count, erythrocyte sedimentation rate [ESR], C-reactive protein [CRP]), and DNI were measured. WBC, CRP, ESR and DNI were higher in APN than in lower UTI (*p* < 0.01). Multiple logistic-regression analyses showed that DNI was a predictive factor for areas of lack of uptake on dimercaptosuccinic acid (DMSA) scans (*P* < 0.01). The area under the receiver operating characteristic (AUC) was also high for DNI (0.622, 95% CI 0.558–0.687, *P* < 0.01) as well as for CRP (0.731, 95% CI 0.673–0.789, *P* < 0.01) for the prediction of DMSA defects. DNI demonstrated the highest area under the ROC curve for diagnosis of VUR (0.620, 95% CI 0.542–0.698, *P* < 0.01). To the best of our knowledge, this is a first study demonstrating that DNI can be used as a diagnostic marker to distinguish APN from lower UTI and function as a diagnostic marker indicative of VUR compared to other conventional markers.

Urinary tract infection (UTI) is one of many bacterial infections common in infants and young children^1^. Early detection of UTI at this age can be difficult due to the fact that the presenting symptoms of UTI such as diarrhea, vomiting, decreased oral intake, and irritability are often vague and non-specific during time of onset^1^,^2^.

More importantly, clinical and laboratory parameters for the early detection of acute pyelonephritis (APN) are required in practice to identify children who could benefit from a Dimercaptosuccinic acid (DMSA) scan. In response, several studies have previously reported various potential non-invasive biomarkers that may be used to identify a systemic inflammatory response including white blood cell (WBC) counts, C-reactive protein (CRP), erythrocyte sedimentation rate (ESR), procalcitonin, cytokines, chemokines, cell surface antigens, and even immunoglobulins and antibodies against NK cells^3^. These biomarkers require analytical methods that need manual supervision or an experienced technician such as enzyme-linked immunoabsorbent assay and flow cytometry. In addition, their clinical usefulness as a diagnostic marker still remains controversial.

During bacterial infection, immature neutrophils such as metamyelocytes, myelocytes and promyelocytes enter the peripheral blood stream from the bone marrow as a result of an enhanced production of granulocytes, a phenomenon known as “shift to the left”^4^. Recently, it has become possible for automated hematologic cell analyzers to provide information on the leukocyte differentials by utilizing the nuclear lobularity of WBC and cytochemical myeloperoxidase (MPO) reaction, and thus, one of our authors has developed the delta neutrophil index (DNI) in the ADVIA2120 (Siemens Healthcare Diagnostics) system which is calculated as the difference between these two parameters^5^,^6^. Recently, several studies have reported that DNI is a more useful marker for predicting mortality than WBC or CRP levels in patients with bacterial sepsis^7^,^8^. However, very few validation studies are published and there has yet to be a report on the clinical usefulness of DNI in children with UTIs.

Therefore, we aimed at an evaluation of the usefulness of DNI as an inflammatory marker in infants and young children with febrile UTI with respect to its potential in identification of APN and vesicoureteral reflux.

## Results

Clinical, laboratory and imaging findings of patients are presented in Table 1. CRP and DNI were significantly higher in patients showing abnormal ultrasonography (USG). Also, the incidence of DMSA abnormalities, VUR and severe VUR was more frequent in the patients with abnormal USG. There were no significant statistical differences in age and sex between patients with DMSA abnormalities (APN group) and those without (lower UTI group). Conventional inflammatory markers such as WBC counts, ESR, CRP levels were significantly higher in the APN group than in the lower UTI group. There was also a significant difference between the APN and lower UTI group in terms of DNI. The incidence of VUR and abnormal ultrasonographic findings were more frequent in the APN group than in the lower UTI group.

In patients with VUR, WBC counts, ESR, DNI were significantly higher than those of VUR negative patients, while CRP levels did not differ between the two groups (Table 2). In addition, ESR and DNI were significantly higher in the severe VUR group than in the mild VUR group. In the imaging study, abnormal USG and abnormal DMSA findings were higher in the patients with severe VUR than in mild VUR. There were no significant differences in WBC, hemoglobin (Hb), platelet counts and CRP levels between the two groups.

Multiple logistic regression analyses were also performed to test whether each parameter could be an independent predictor for renal USG and DMSA scan abnormalities or the presence of VUR and severe VUR. We found that only DNI was an independent predictive factor for renal USG abnormalities in UTI patients, while WBC, ESR and CRP were not (Table 3). In addition, DNI and CRP were independent predictive factors for DMSA scan abnormalities, while WBC and ESR were not. More importantly, only DNI was an independent predictive factor for the presence of severe VUR, while WBC, ESR and CRP were not.

**Figure 1:**
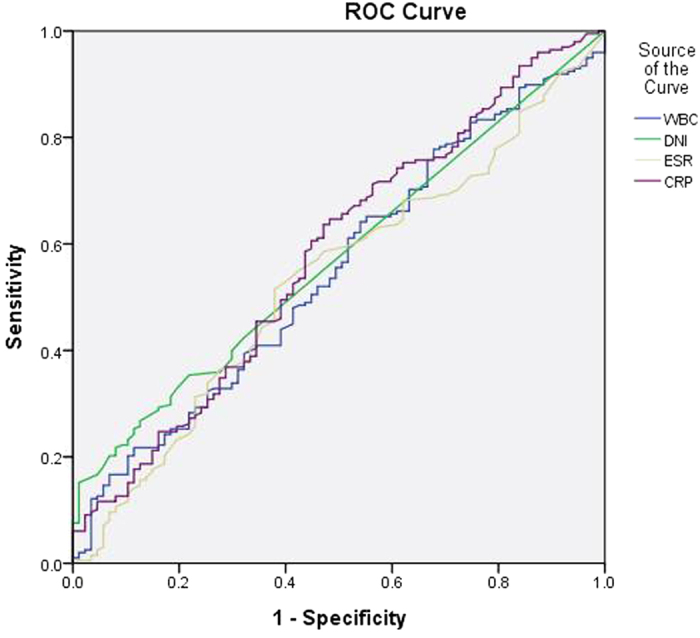
Receiver operating characteristic (ROC) curve of DNI and other inflammatory markers for the prediction of USG abnormalities. DNI: delta neutrophil index, USG: ultrasonography, WBC: white blood cell, ESR: erythrocyte sedimentation rate, CRP: C-reactive protein.

**Figure 2:**
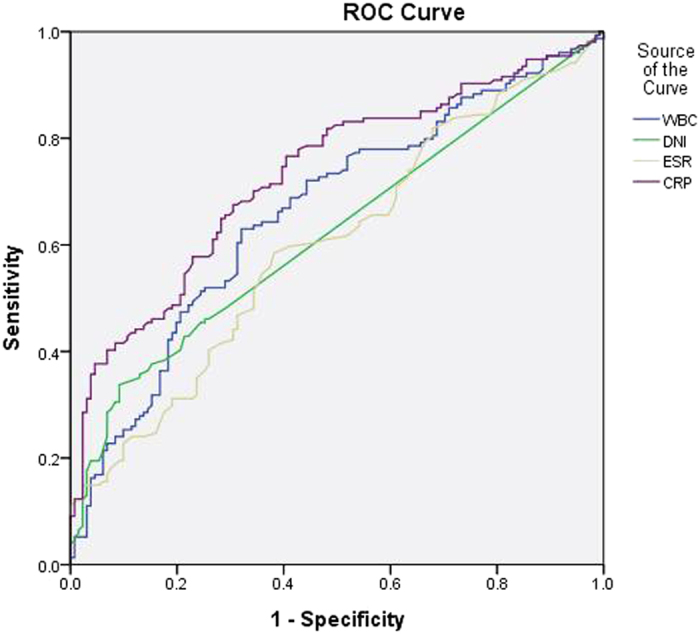
Receiver operating characteristic (ROC) curve of DNI and other inflammatory markers for the prediction of DMSA abnormalities. DNI: delta neutrophil index, DMSA: dimercaptosuccinic acid, WBC: white blood cell, ESR: erythrocyte sedimentation rate, CRP: C-reactive protein.

**Figure 3:**
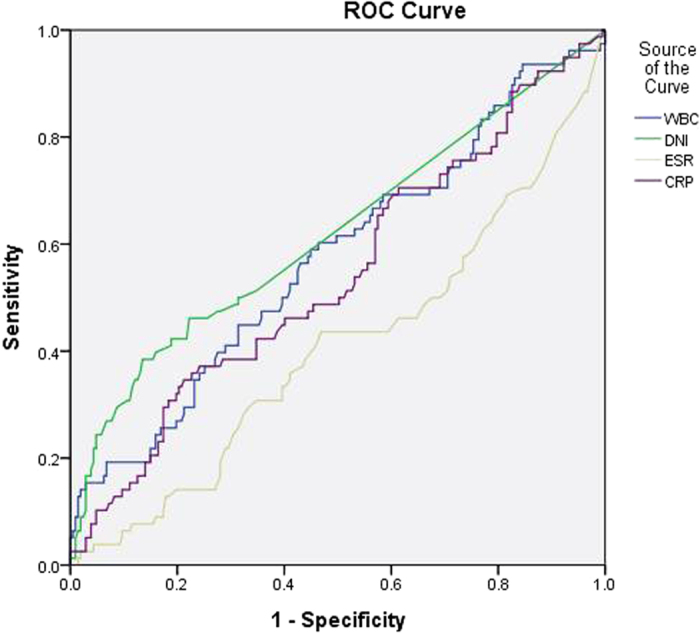
Receiver operating characteristic (ROC) curve of DNI and other inflammatory markers for the prediction of VUR. DNI: delta neutrophil index, VUR: vesicoureteral reflux, WBC: white blood cell, ESR: erythrocyte sedimentation rate, CRP: C-reactive protein.

**Figure 4:**
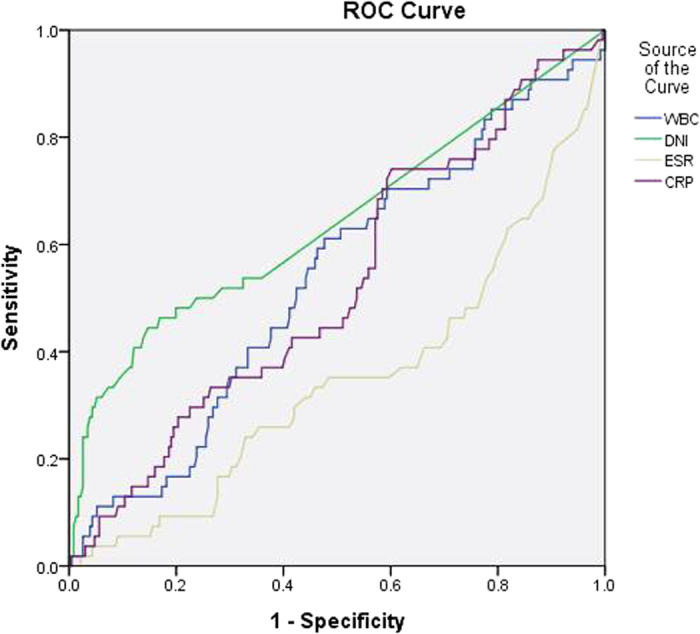
Receiver operating characteristic (ROC) curve of DNI and other inflammatory markers for the prediction of severe VUR. DNI: delta neutrophil index, VUR: vesicoureteral reflux, WBC: white blood cell, ESR: erythrocyte sedimentation rate, CRP: C-reactive protein.

The diagnostic properties of the various inflammatory markers were examined for their predictive power regarding renal USG and DMSA scan abnormalities, and the presence of both severe and non-severe VUR using receiver operating characteristic (ROC) curves (Table 4). Regarding the predictive power for renal ultrasonography abnormalities, the median area under the ROC curve (AUC) was 0.549 for WBC (*P* = 0.18), 0.533 for ESR (*p* = 0.37), 0.579 for CRP (*P* = 0.03) and 0.573 for DNI (*P* = 0.05). These results show that the diagnostic property represented by AUC of all various inflammatory markers was not high for the prediction of renal USG abnormalities. Regarding the predictive power for DMSA scan abnormalities, the median AUC was 0.663 for WBC (*P* < 0.01), 0.622 for DNI (*P* < 0.01), 0.600 for ESR (*P* < 0.01) and 0.731 for CRP (*P* < 0.01). The diagnostic property of DNI was not higher than CRP for the prediction of DMSA scan abnormalities. However, the median AUC of DNI was highest among all other inflammatory markers for prediction of the presence of VUR (AUC 0.620, 95% CI 0.542–0.698, *P* < 0.01) or severe VUR (AUC 0.642, 95% CI 0.549–0.734, *P* = 0.001) (Figs 1, 2, 3 and 4).

The sensitivity, specificity, positive predictive values (PPV), negative predictive values (NPV), positive likelihood ratios (PLR), and negative likelihood ratios (NLR) of the DNI were determined under different cut-off values. DNI showed a moderate specificity and low sensitivity for the prediction of renal USG and DMSA scan abnormalities and the presence of VUR or severe VUR (Table 5 and Supplementary Figure S1). In addition, serum DNI did not correlate with WBC and CRP (Table 6).

## Discussion

This study is the first to show that DNI, which reflects the number of circulating granulocyte precursors in the blood, can be an effective marker to differentiate APN from lower UTI in young children. Although UTI can be localized to the bladder (lower UTI), infection can also spread to the renal parenchyma and cause APN which is associated with the formation of a renal scar and increased risk of hypertension and chronic kidney disease (CKD)^9^,^10^. DMSA scan is considered to be the current gold standard of detecting and evaluating the extent of acute renal lesions, but it exposes to radiation and sedation is necessary^11^,^12^. Therefore, the selection of children who need DMSA scan has been a constant issue of debate^13^.

Recently, however, the National Institute for Health and Care Excellence (NICE) clinical guideline on urinary tract infections discouraged routine imaging of all children with first febrile UTI. According to the most updated guidelines, DMSA is recommended only in children less than 3 years of age with atypical or recurrent UTI^14^. Furthermore, American Academy of Pediatrics guidelines also discourage prescribing routine imaging studies for first UTI in children aged between 2 and 24 months^15^. Thus, DMSA is no longer a routine investigation for first febrile UTI, as evidence continues to support the fact that the yield of actionable findings from imaging is relatively low.

Until now, the commonly used laboratory inflammatory markers are WBC counts, ESR, CRP, and procalcitonin^16^,^17^,^18^,^19^. But, neither clinical parameters nor these inflammatory markers are able to reliably differentiate APN from lower UTI in infants and young children due to the lack of specificity^20^. Interestingly, in previous studies, the proportion of immature granulocytes better correlated with bacterial infection compared to the total WBC counts^21^. Consistent with this finding, the level of immature granulocytes was also suggested as a predictor of neonatal sepsis^22^.

Such stronger association of immature granulocytes percentage with infection is presumed to have been demonstrated in these studies because immature granulocytes are massively recruited from bone marrow and enter into circulation as part of the innate immune response. In fact, during the early hyperdynamic phase of infection, a proinflammatory state is heavily mediated by neutrophils, macrophages and monocytes with release of inflammatory cytokines, such as tumor necrosis factor-α (TNF-α) and interleukin-1 and -6. During this phase, the systemic inflammatory response suppresses neutrophil apoptosis and thus augments neutrophil-mediated killing as part of the innate response while simultaneously increasing lymphocyte apoptosis in the thymus and spleen^4^,^23^.

In light of this phenomenon, one of our authors developed a novel value known as the delta neutrophil index (DNI) which is calculated by subtracting the leukocyte subfraction counted in the nuclear lobularity channel from the leukocyte subfraction obtained through the MPO cytochemical reaction. This value correlates with the fraction of immature granulocytes in the peripheral blood under manual counting and by using automated hematologic cell analyzer during a routine complete blood count (CBC) test. The value can be calculated easily. More importantly, this automated process allows for a faster turnaround time, which has been reported to be a crucial factor in improving the quality of patient care and patient outcome, time until diagnosis, and in developing appropriate treatment plans while also significantly reducing laboratory cost^24^,^25^. Furthermore, this advantage becomes more prominent during real-time ordering of laboratory tests of patients with a severe medical condition needing early intervention such as sepsis. With regard to the diagnostic validity of this value, results from a previous study have shown that, compared to WBC counts or CRP, the DNI value is in fact a more useful early marker for disease severity in critically ill patients with sepsis^26^. Furthermore, several other studies have also revealed that the DNI can help identify infections such as bacteremia and early sepsis in immunocompetent children^27^, patients with adult onset Still’s disease (AOSD)^28^, patients whose blood culture samples are contaminated^29^, and febrile systemic lupus erythematosus patients^30^. While many studies have proven that the DNI can be potentially useful in the early diagnosis of systemic infection such as sepsis, whether this phenomenon also holds true for localized infection such as APN.

Interestingly, our univariate analysis indicated statistically significant differences in the mean DNI and CRP values between patients with USG and DMSA abnormalities and those without abnormalities. The same univariate analysis revealed significant difference in the WBC count and ESR but this difference was only significant in the comparison between patients with DMSA abnormalities and those with normal DMSA, thus indicating that the DNI value better discriminates patients with abnormal USG compared to WBC count and ESR similar to CRP.

Similarly, univariate analysis of patients with VUR revealed that the DNI and ESR are able to discriminate patients with VUR from those without while at the same time being able to discriminate severe VUR from mild VUR. This result shows that an increase in DNI not only can be used to identify patients with VUR and may predict the severity of the disease based on the level of increase. Considering that ESR is able to differentiate patients with VUR while CRP can discriminate patients with either abnormal USG or DMSA findings in the absence of DNI, neither of these inflammatory markers is able to give complete information in formulating a clinical impression of a patient. Moreover, both markers would need to be measured to supplement each other’s lack of clinical information. However, with the DNI, both information regarding the probability of abnormal USG and DMSA scan results along with the probability of presence and severity of VUR may be obtained with one test.

Consistent with these findings, our multiple logistic regression analysis indicates that DNI value is the only marker that is statistically significant in predicting USG and DMSA abnormalities and both presence of VUR and severe VUR. All other markers were found to be significant in predicting one of four groups: the WBC count and ESR for the presence of VUR, and CRP for the presence of DMSA abnormalities. In the multiple logistic regression analysis, the only marker that was statistically significant for USG abnormalities and severe VUR was the DNI.

In addition, while the AUC of DNI in patients with USG abnormalities was borderline significant (*P* = 0.05) it was nearly comparable to that of CRP, both of which being much more significant than either the WBC count and ESR. Admittedly, an AUC value less than 0.6 is not indicative of an acceptable accuracy and thus the AUC of DNI in patients with USG abnormalities may signal poor accuracy as a diagnostic tool. However, compared to the AUC of other markers especially the WBC count and ESR, the DNI holds much better accuracy almost equivalent to that of the CRP. Also, the DNI is the only marker with a statistically significant AUC (*P* value < 0.05) greater than 0.6 for all other categories including patients with DMSA abnormalities, VUR and severe VUR. Furthermore, in the case of patients with VUR and severe VUR, the DNI showed the highest and acceptable AUC value, thereby showing that it is the only marker with noteworthy accuracy as a diagnostic tool.

Our study also indicates that the DNI while having less than 50% sensitivities in all criterion values in all four categories, still has a moderate to high specificity ranging from 70 to 90%. Interestingly, such low sensitivity of DNI in UTI differs from the reports of high sensitivities ranging from 82% to 95% by previous studies^27^,^29^. However, this difference is most likely attributed to the fact that these past studies evaluated the DNI’s accuracy in patients with bacteremia and sepsis, both of which are considered as systemic infection unlike APN and the lower UTI, which is a localized infection that was analyzed in our study. In fact, in a study^31^ that analyzed the mean DNI in patients with bacteremia, sepsis and septic shock, the mean DNI for the above three conditions were 8.7%, 24.2% and 35.6%, all of which are much higher than the 1.3% and 1.5% observed in APN patients with abnormal USG and DMSA in our study.

In regards to specificity, as expected, with an increase in the criterion or cut off values, the DNI specificities also increased due to reduced false positives. However, even at the lowest criterion, the DNI specificities range from 71% to 76%, which is consistent with the specificity of 75% reported by Lee *et al*.^29^. However, previous studies^27^,^28^,^30^ have also reported higher DNI specificities ranging from 84 to 95%. Similar to the sensitivities, the discrepancy between the specificity of DNI of our result with those of past studies is thought to have occurred due to the localization of the infection in the case of UTI. However, even with a specificity of ~70% in the case of APN, it is important to note that the positive likelihood ratio (LR) ranges from 1.33 to 3.78 and thus considering a pretest probability of 36.1%, which is the prevalence of VUR and severe VUR in our study, a posttest probability of at least 48.0% can be expected with this probability being greater with higher DNI value. Overall, however, such moderate specificity in presence of low sensitivity indicates that the DNI may be more suitable as a diagnostic predictive factor rather than a preliminary marker for screening in the case of APN.

Lastly, results of the correlation analysis indicates that while the WBC, ESR and CRP correlate well with each other, the DNI does not correlate with the rest of the inflammatory markers. This is expected considering the fact that the proliferation and introduction of immature neutrophils into the circulation occurs during the earliest phase of stress or infection before the total WBC count, ESR and CRP increases. The release and synthesis of procalcitonin by thyroid C cells and various neuroendocrine tissues and CRP by the liver, although they have been reported to strongly correlate with the severity and mortality of infection such as sepsis, are presumed to play less important roles in an acute inflammation^32^,^33^. In such respect, DNI can be considered to be a better marker in identifying the early phase of an infection, making translation to faster intervention possible.

To summarize, this study is the first exploring the potential of using DNI as an adjunct diagnostic marker to distinguish APN from lower UTI. Furthermore, the results of our study indicate that the DNI may better serve in predicting VUR in young children compared to the other currently available conventional markers. However, due to the limitation of small study population and retrospective design of our study, further studies may still be needed to evaluate the relationship between the DNI and proinflammatory cytokines.

## Methods

### Patients and inclusion criteria

We retrospectively studied 288 infants less than 12 months old or young children who were hospitalized with a first febrile UTI in a 5-year period in Yonsei University Severance hospital from January 2010 to December 2014. The diagnosis of a febrile UTI was based on the following criteria: (1) fever of more than 38 °C, (2) pyuria (≥5 WBC counts per high-power field), (3) growth of a single organism (≥100,000 colony-forming units/mL) in urine collected from sterile bag or transurethral catheterized specimen, (4) no previous history of UTI and no other coincidental infections and (5) imaging studies, including renal USG, DMSA, and voiding cystourethrogram (VCUG) were performed.

These patients are overlapped in our previous cohort^2^. Empirical antibiotics with intravenous ceftriaxone or cefotaxime were initiated in all patients for 3–7 days, which were changed to oral antibiotics after 7 days. Total duration of treatment was 14 days. Patients were divided into the two groups according to the presence of abnormalities on renal USG and DMSA scans or the presence of VUR or severe VUR.

### Laboratory examinations and DNI

Before initiating antibiotic treatment, blood was sampled for laboratory investigations, including WBC count, ESR, and CRP at the time of visiting hospital. Peripheral venous blood samples were collected by antecubital venepuncture into Vacutainer tubes (Becton Dickinson™, Rutherford, NJ) containing tripotassium ethylenediaminetetraacetic acid (EDTA) and were immediately transported to the chemical laboratory department. CBC studies were done within one hour of blood sampling and we followed a standardized protocol. DNI is included as a part of the routine CBC tests at our institution. DNI calculation was carried out using an automatic cell analyzer (ADVIA 2120 Hematology System, Siemens Healthcare Diagnostics, Forchheim, Germany)^5^. After lysis of red blood cells, cell size and stain intensity were measured by the tungsten-halogen-based optical system of the MPO channel to count and differentiate granulocytes, lymphocytes, and monocytes based on their size and MPO content. Next, cells were counted and classified according to the size, lobularity, and nuclear density, using the laser diode-based optical system of the lobularity nuclear density channel. The DNI value was calculated using the following formula: DNI = [the neutrophil subfraction and the eosinophil subfraction measured in the MPO channel by the cytochemical MPO reaction]—[the polymorphonuclear neutrophil (PMN) subfraction measured in the nuclear lobularity channel by the reflected light beam]. The unit of DNI value was expressed as percentage (%). CRP levels were measured by the latex-enhanced turbidimetric assay method using a Hitachi 7600 P module (Hitachi, Japan). ESR levels were measured by the TEST 1 (Alifax, Padova, Italy). Strict quality control procedures were adopted.

### Imaging Studies

Renal USG was performed in all patients to detect anomalies in kidney and urinary tracts. DMSA scans were also performed within the first 5 days after admission to detect any renal parenchymal involvement which was considered to be positive when a focal, multifocal, or diffuse decrease or absence of DMSA uptake was noted. Presence of renal parenchymal involvement on DMSA scan was determined by one nuclear medicine radiologist who was blinded to this study. VCUG was performed 1–2 weeks after the completion of UTI antibiotic treatment and VUR was diagnosed according to the International Reflux Study defined as the retrograde passage of urine from the bladder into ureters or kidney. Mild VUR was defined as VUR grade 1, 2 and severe VUR as VUR grade 3–5. Parents were informed of the potential benefits and risks of DMSA scan and VCUG and their consents were received before the examinations.

### Statistical methods

Statistical analyses were performed, using the SPSS for Windows version 18.0 (SPSS Inc., Chicago, Illinois, USA) and MedCalc version 15.8 (MedCalc Software, Belgium). The independent t-test was used for continuous variables and expressed as mean ± standard deviation (SD). Chi-square test was used to analyze categorical variables. Correlation analysis was also carried out to determine the relationship between two variables by Pearson correlation. Multiple logistic regression analysis was used to find independent predictive factors for renal USG and DMSA scan abnormalities or the presence of VUR and severe VUR. To establish the predictive value of the parameters for renal USG and DMSA scan abnormalities or the presence of VUR, receiver operating characteristic (ROC) curves were plotted for WBC, ESR, CRP and DNI. The diagnostic values of each cutoff point, including sensitivity, specificity, PPV, NPV and the likelihood ratio for a positive result, were all calculated. All differences were considered statistically significant at a P value < 0.05.

### Ethics statement

The Institutional Review Board and Research Ethics Committee of Yonsei University Severance Hospital approved this study. We were given exemption from getting informed consents by the IRB because the present study was a retrospective study, personal identifiers were completely removed, and the data were analyzed anonymously. Our study was conducted according to the ethical standards laid down in the 1964 Declaration of Helsinki and its later amendments.

## Additional Information

**How to cite this article:** Lee, J. W. *et al*. The value of delta neutrophil index in young infants with febrile urinary tract infection. *Sci. Rep.* **7**, 41265; doi: 10.1038/srep41265 (2017).

**Publisher's note:** Springer Nature remains neutral with regard to jurisdictional claims in published maps and institutional affiliations.

## Supplementary Material

Supplementary Information

## Figures and Tables

**Table 1:** Comparison of variables between patients with and without abnormalities in USG and DMSA scans.

	Abnormal USG (n = 198)	Normal USG (n = 90)	*P* value	DMSA abnormalities (n = 155)	Normal DMSA (n = 133)	*P* value
Age (months)	4.6 ± 3.5	4.9 ± 3.9	0.47	4.3 ± 2.5	4.1 ± 2.1	0.53
Gender (Male/Female)	149/49	60/30	0.15	112/43	97/36	0.90
**Laboratory findings**						
WBC (/mm^3^)	16,201.3 ± 6,312.2	14,931.3 ± 5,593.1	0.09	17,338 ± 6,290	14,045 ± 5,363	<0.01
Hb (g/L)	11.0 ± 1.0	11.0 ± 1.0	0.90	10.9 ± 1.0	11.1 ± 1.0	0.15
Platelet (x10^3^/μL)	456.3 ± 152.4	454.7 ± 137.2	0.93	463.0 ± 159.4	455.0 ± 132.5	0.64
ESR (mm/h)	40.0 ± 29.2	37.3 ± 29.1	0.45	43.9 ± 31.4	32.6 ± 24.4	<0.01
CRP (mg/L)	58.8 ± 63.6	41.7 ± 37.8	<0.01	71.8 ± 68.1	21.6 ± 27.0	<0.01
DNI (%)	1.3 ± 2.7	0.5 ± 0.9	<0.01	1.5 ± 2.9	0.5 ± 1.1	<0.01
**Imaging findings**						
USG abnormalities	—	—	—	117 (59.1%)	38 (42.2%)	<0.01
DMSA abnormalities	117/198 (59.1%)	38/90 (42.2%)	0.01	—	—	—
VUR	64/198 (32.3%)	14/90 (15.6%)	<0.01	65/155 (41.9%)	13/133 (9.8%)	<0.01
Severe VUR	49/64 (76.6%)	5/14 (35.7%)	<0.01	49/65 (75.4%)	5/13 (38.5%)	0.01

USG: ultrasonography, DMSA: dimercaptosuccinic acid, WBC: white blood cell, Hb: Hemoglobin, ESR: erythrocyte sedimentation rate, CRP: C-reactive protein, DNI: delta neutrophil index, VUR: vesicoureteral reflux.

**Table 2:** Comparison of variables between patients with and without VUR and severe VUR.

	VUR (n = 78)	No VUR (n = 210)	*P* value	Severe VUR (n = 54)	Mild VUR (n = 24)	*P* value
Age (months)	4.3 ± 2.5	4.1 ± 2.3	0.69	3.9 ± 2.4	5.1 ± 2.3	0.03
Gender (Male/Female)	59/19	150/60	0.55	43/11	16/8	0.25
**Laboratory findings**						
WBC (/mm^3^)	17,352.8 ± 7,209.4	15,247.4 ± 5,539.2	0.02	16,419.4 ± 6,508.0	19,452.9 ± 8,352.1	0.12
Hb (g/L)	11.1 ± 1.1	10.9 ± 0.9	0.10	11.1 ± 1.1	11.3 ± 1.1	0.53
Platelet (x10^3^/μL)	452.7 ± 154.4	461.7 ± 145.1	0.65	437.3 ± 152.1	487.4 ± 156.6	0.18
ESR (mm/h)	32.5 ± 28.4	41.0 ± 28.8	0.02	21.8 ± 27.3	43.1 ± 28.6	0.02
CRP (mg/L)	64.5 ± 80.6	49.1 ± 28.8	0.11	57.1 ± 56.1	81.2 ± 118.6	0.22
DNI (%)	1.9 ± 3.3	0.7 ± 1.6	<0.01	2.4 ± 3.8	0.9 ± 1.3	<0.01
**Imaging findings**						
USG abnormalities	64/78 (82.1%)	134/210 (63.8%)	<0.01	49/54 (90.7%)	15/24 (62.5%)	<0.01
DMSA abnormalities	65/78 (83.3%)	90/210 (42.9%)	<0.01	49/54 (90.7%)	16/24 (66.7%)	0.01

VUR: vesicoureteral reflux, WBC: white blood cell, Hb: Hemoglobin, ESR: erythrocyte sedimentation rate, CRP: C-reactive protein, DNI: delta neutrophil index, USG: ultrasonography, DMSA: dimercaptosuccinic acid.

**Table 3:** Multiple logistic regression analysis of laboratory parameters for the prediction of USG and DMSA abnormalities and VUR or severe VUR.

	USG abnormalities		DMSA abnormalities		VUR		Severe VUR	
	OR (95% CI)/B	*P* value	OR (95% CI)/B	*P* value	OR (95% CI)/B	*P* value	OR (95% CI)/B	*P* value
WBC	1.000 (1.000–1.000)/0.000	0.98	1.000 (1.000–1.000)/0.000	0.25	1.000 (1.000–1.000)/0.000	0.02	1.000 (1.000–1.000)/0.000	0.16
ESR	0.998 (0.987–1.009)/−0.002	0.72	0.994 (0.981–1.006)/−0.006	0.29	0.977 (0.965–0.990)/−0.023	<0.01	0.988 (0.967–1.008)/−0.012	0.23
CRP	1.005 (0.999–1.011)/0.005	0.09	1.024 (1.015–1.032)/0.023	0.00	1.007 (1.000–1.014)/0.007	0.05	0.999 (0.993–1.006)/−0.001	0.82
DNI	1.297 (1.055–1.595)/0.260	0.01	1.337 (1.106–1.616)/0.290	<0.01	1.215 (1.065–1.386)/0.195	<0.01	1.430 (1.045–1.956)/0.357	0.02

USG: ultrasonography, DMSA: dimercaptosuccinic acid, VUR: vesicoureteral reflux, OR: odds ratio, CI: confidence interval, B: regression coefficients, WBC: white blood cell, ESR: erythrocyte sedimentation rate, CRP: C-reactive protein, DNI: delta neutrophil index.

**Table 4:** AUC values from receiver operating characteristic (ROC) curve of DNI and other inflammatory markers for the prediction of USG and DMSA abnormalities and VUR or severe VUR.

	USG abnormalities		DMSA abnormalities		VUR		Severe VUR	
	AUC (95% CI)	*P* value	AUC (95% CI)	*P* value	AUC (95% CI)	*P* value	AUC (95% CI)	*P* value
WBC	0.549 (0.478–0.621)	0.18	0.663 (0.600–0.726)	<0.01	0.574 (0.498–0.651)	0.04	0.535 (0.450–0.620)	0.43
ESR	0.533 (0.462–0.605)	0.37	0.600 (0.535–0.666)	<0.01	0.406 (0.330–0.482)	0.04	0.350 (0.264–0.435)	<0.01
CRP	0.579 (0.507–0.652)	0.03	0.731 (0.673–0.789)	<0.01	0.542 (0.465–0.618)	0.27	0.525 (0.440–0.610)	0.57
DNI	0.573 (0.504–0.642)	0.05	0.622 (0.558–0.687)	<0.01	0.620 (0.542–0.698)	<0.01	0.642 (0.549–0.734)	<0.01

AUC: Area under the curve, DNI: delta neutrophil index, USG: ultrasonography, DMSA: dimercaptosuccinic acid, VUR: vesicoureteral reflux, CI: confidence interval, WBC: white blood cell, ESR: erythrocyte sedimentation rate, CRP: C-reactive protein.

**Table 5:** Diagnostic accuracy of DNI for the prediction of renal USG and DMSA abnormalities and VUR or severe VUR.

Variable	Criterion	Sensitivity	Specificity	Positive predictive value	Negative predictive value	Positive LR	Negative LR
USG abnormalities	>0.4	38.38 (31.6–45.5)	71.11 (60.6–80.2)	12.9 (6.6–21.8)	91.2 (86.4–94.7)	1.33 (0.9–1.9)	0.87 (0.7–1.0)
	>0.9	31.31 (24.9–38.3)	82.22 (72.7–89.5)	16.4 (7.8–28.8)	91.5 (87.2–94.8)	1.76 (1.1–2.9)	1.33 (0.9–1.9)
	>1.3*	26.77 (20.7–33.5)	87.78 (79.2–93.7)	19.6 (8.7–35.3)	91.5 (87.3–94.7)	2.19 (1.2–4.0)	0.83 (0.7–0.9)
DMSA abnormalities	>0.4	45.16 (37.2–53.3)	75.94 (67.8–82.9)	17.3 (9.5–27.7)	92.6 (88.2–95.7)	1.88 (1.3–2.7)	0.72 (0.6–0.9)
	>0.9	37.42 (29.8–45.5)	84.96 (77.7–90.6)	21.7 (11.2–35.6)	92.4 (88.3–95.5)	2.49 (1.6–3.9)	0.74 (0.6–0.8)
	>1.3*	33.55 (26.2–41.6)	90.98 (84.8–95.3)	29.2 (14.8–47.6)	92.5 (88.5–95.4)	3.72 (2.1–6.7)	0.73 (0.6–0.8)
VUR	>0.4	48.72 (37.2–53.3)	75.94 (67.8–82.9)	15.1 (8.5–24.0)	92.4 (87.8–95.7)	1.60 (1.2–2.2)	0.74 (0.6–0.9)
	>0.9	42.31 (31.2–54.0)	78.57 (72.4–83.9)	18.0 (9.7–29.2)	92.5 (88.1–95.6)	1.97 (1.4–2.8)	0.73 (0.6–0.9)
	>1.3	39.74 (28.8–51.5)	84.29 (78.6–88.9)	21.9 (11.7–35.6)	92.6 (88.5–95.6)	2.53 (1.7–3.8)	0.71 (0.6–0.9)
	>1.8*	38.46 (27.7–50.2)	86.67 (81.3–91.0)	24.3 (12.8–39.2)	92.7 (88.7–95.6)	2.88 (1.8–4.5)	0.71 (0.6–0.9)
Severe VUR	>0.9	48.15 (34.3–62.2)	70.83 (48.9–87.4)	15.5 (4.2–35.9)	92.5 (81.9–97.9)	1.65 (0.8–3.3)	0.73 (0.5–1.1)
	>1.8*	44.4 (30.9–58.6)	85.47 (80.3–89.7)	25.4 (14.2–39.6)	93.3 (89.3–96.1)	3.06 (2.0–4.7)	0.65 (0.5–0.8)
	>2.3	40.74 (27.6–55.0)	83.33 (62.6–95.3)	21.4 (4.9–49.7)	92.7 (83.3–97.7)	2.44 (0.9–6.3)	0.71 (0.5–0.9)
	>3.0	31.48 (19.5–45.6)	91.67 (73.0–99.0)	29.6 (5.1–68.4)	92.3 (83.4–97.3)	3.78 (0.9–15.1)	0.75 (0.6–0.9)

DNI: delta neutrophil index, USG: ultrasonography, DMSA: dimercaptosuccinic acid, VUR: vesicoureteral reflux, LR: likelihood ratio, ^*^Recommended cut-off value by Youden index.

**Table 6:** Correlations between WBC, ESR, C-reactive protein and DNI in patients with UTI.

	WBC (/mm^3^)	ESR (mm/hr)	CRP (mg/L)	DNI (%)
WBC (/mm^3^)	—	<0.01^*^ (0.345)^**^	<0.01 (0.449)	0.14 (0.087)
ESR (mm/hr)	<0.01 (0.345)	—	<0.01 (0.494)	0.50 (−0.040)
CRP (mg/L)	<0.01 (0.449)	<0.01 (0.494)	—	0.09 (0.099)
DNI (%)	0.14 (0.087)	0.50 (−0.040)	0.09 (0.099)	—

UTI: Urinary tract infection, WBC: white blood cell, ESR: erythrocyte sedimentation rate, CRP: C-reactive protein, DNI: delta neutrophil index, *P-value, **Correlation coefficient.
