# Supplementary material for: The value of delta neutrophil index in young infants with febrile urinary tract infection
Source: Sci Rep. 2017 Feb 7;7:41265. doi: 10.1038/srep41265 (PMC5294644; doi:10.1038/srep41265)

**Supplementary figures**

**The value of delta neutrophil index in young children with febrile urinary tract infection**

Jung Won Lee^1,*^, Seong Heon Kim^2,*^, Se Jin Park^3,*^, Keum Hwa Lee^4,5,*^, Jae Hyon Park^6,*^, Andreas Kronbichler^7^, Michael Eisenhut^8^, Ji Hong Kim^4^, Jong Wook Lee^9,10^ and Jae Il Shin^4,5,11^

^1^Department of Pediatrics, Ewha Womans University School of Medicine, Seoul, Korea

^2^Department of Pediatrics, Pusan National University Children’s Hospital, Yangsan, Korea

^3^Department of Pediatrics, Geoje Children's Hospital, Geoje, Korea

^4^Department of Pediatrics, Yonsei University College of Medicine, Seoul, Korea

^5^Department of Pediatric Nephrology, Severance Children’s Hospital, Seoul, Korea

^6^Yonsei University College of Medicine, Seoul, Korea

^7^Medical University Innsbruck, Department of Internal Medicine IV (Nephrology and Hypertension), Innsbruck, Austria

^8^Luton & Dunstable University Hospital NHS Foundation Trust, Luton, United Kingdom

^9^Department of Laboratory Medicine, Jincheon Sungmo Hosipital, Jincheon, Korea

^10^Research Institute of Bacterial Resistance, Yonsei University college medicine, Seoul, Korea

^11^Institute of Kidney Disease Research, Yonsei University College of Medicine, Seoul, Korea

*Jung Won Lee, Seong Heon Kim, Se Jin Park, Keum Hwa Lee and Jae Hyon Park contributed equally to this work.

**Supplementary figure S1. Receiver operating characteristic (ROC) curve of DNI for the prediction of (1) USG abnormalities, (2) DMSA abnormalities, (3) the presence of VUR and (4) the presence of severe VUR** DNI: delta neutrophil index, USG: ultrasonography, DMSA: dimercaptosuccinic acid, VUR: vesicoureteral reflux

**(1)**


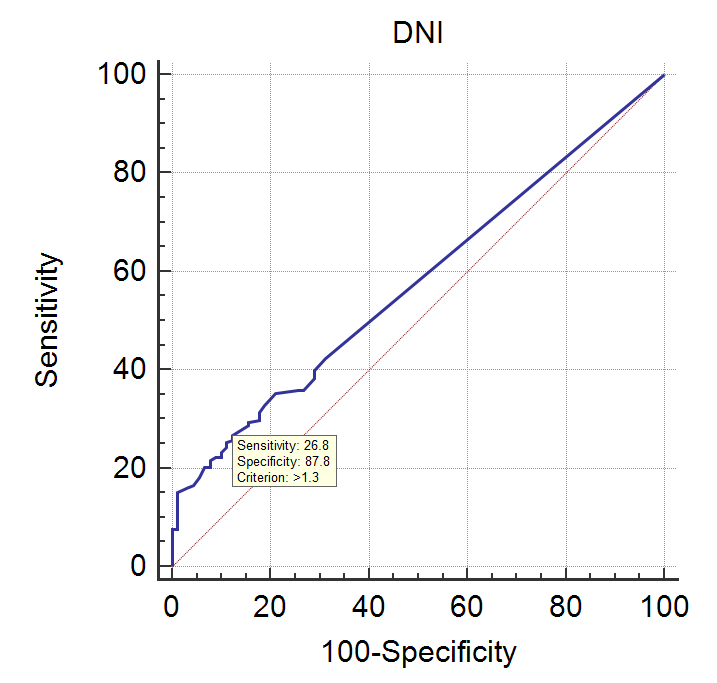


**(2)**


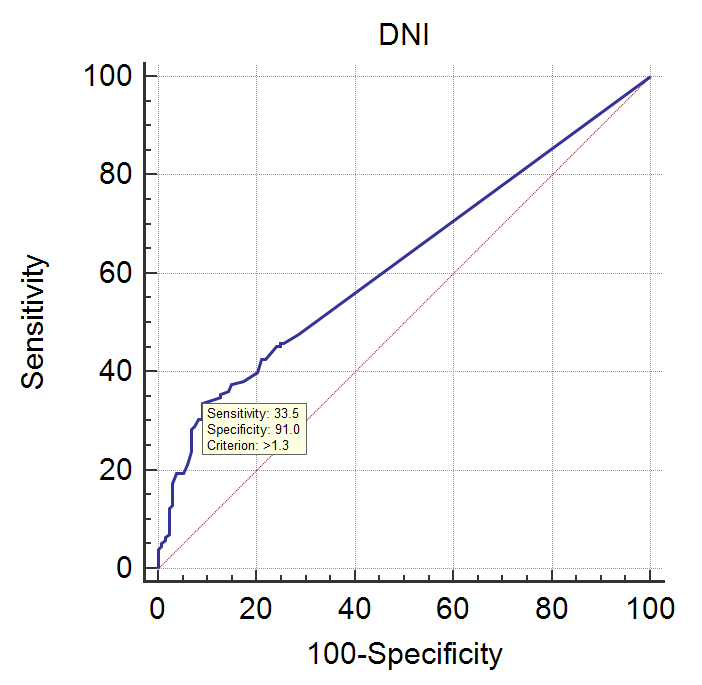


**(3)**


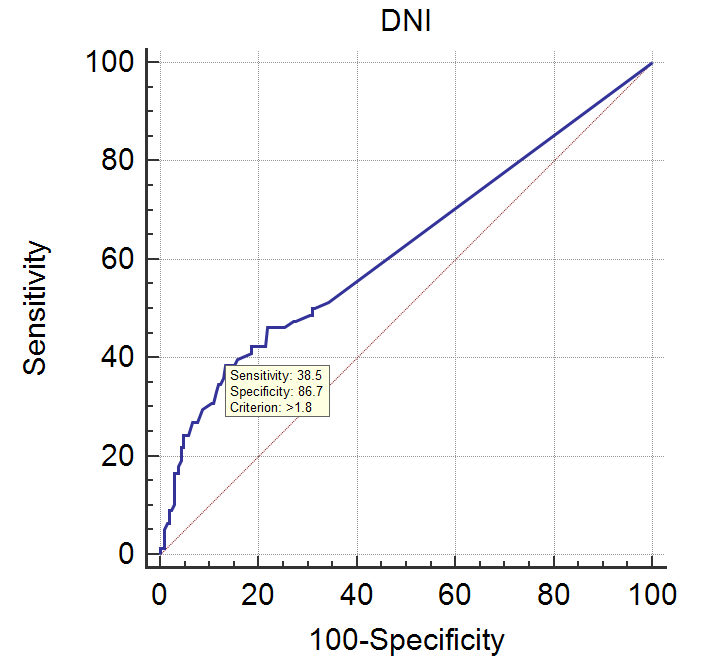


**(4)**


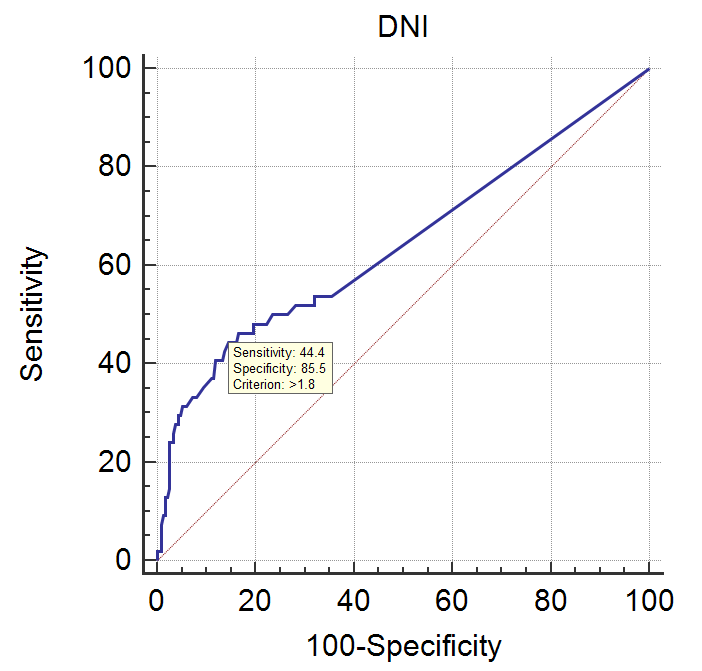

Supplement: Supplementary Information [file srep41265-s1.doc]
